# Knowledge, Attitude and Practice of Nurses Regarding Organ Donation

**DOI:** 10.5539/gjhs.v7n6p129

**Published:** 2015-04-03

**Authors:** Mohadese Babaie, Mahdi Hosseini, Jalaleddin Hamissi, Zahra Hamissi

**Affiliations:** 1 Department of Nursing and Midwifery, Shahid Beheshti University of Medical Sciences, Tehran, Iran; 2 Tehran Heart Center, Tehran University of Medical Sciences, Tehran, Iran; 3 Department of Periodontics & Preventive Dentistry, College of Dentistry, Qazvin University of Medical Sciences, Qazvin, Iran; 4 Dental Student College of Dentistry, Shahied Behesti University of Medical Sciences, Teheran, Iran

**Keywords:** organ donation, knowledge, attitude, nursing practice

## Abstract

**Introduction:**

Treatment team charged to help patients and their family making decision about donate organs in the final stage of life. Hence, their knowledge and attitude is important to plan of increasing the rate of organ donation.

**Materials and Methods:**

About 150 nurses recruited in this cross-sectional study randomly. After taking informed consent, questionnaires were filled. The data collection tool was a multipart questionnaire including demographic information, 18 questions about attitude and practice and 15 question about knowledge toward organ donation. Data were analyzed by SPSS software using K-squire, Pearson correlation test, T-test, variance analyze on 95% confidence interval.

**Results:**

Most of participants (76%) were 25-44 years old. About 81.3% of them were female (n=122). The attitude average score between males and females was 85.25±35.61 and 70.37±46.53, respectively. The practice average score in females was 34.43±47.71 and between males was 29.63±46.53. The knowledge average scores were 50.60±16.19 and 56.54±17.48 for two groups (p>0.05). The knowledge average scores between different age groups was significant (p<0.05). There was a direct and significant relation between attitude and practice (r= +0.33, p<0.05), attitude and Factors influencing attitude and practice (r= 0.866, p<0.05), but the relation between attitude and knowledge was indirect and significant (r= -0.183, p<0.05).

**Conclusions:**

Since the medical team are most important adviser for promote activities related to organ donation, it seems that educational curriculum and facilities should applied to enhance attitude and behavior favorable change of personnel towards this issue.

## 1. Introduction

Nowadays, Organ donation became a typical procedure to save life and improve the lives of the patients that have a chance to survive (Topic, Brkljacic, & Grahovac, 2006).

Organ donation is a standard method of treatment in various diseases; however, the number of patients on the waiting list is more than organs that can be donated (Stadlbauer et al., 2013). This procedures saves the life of millions of people in the world and is a process in which an organ or part of that grafts to another person (Ramadurg & Gupta, 2014).

The process of organ donation depends on its presentation and access to families to obtain their consent (Douville, Godin, & Vézina-Im, 2014). Various factors play a role in the family decision about this issue, For example, Positive beliefs and attitudes about organ donation, Families awareness of the deceased for organ donation, having donation card and expressed interest in this area (Siminoff, Gordon, Hewlett & Arnold, 2001).

Researches shows that positive and negative attitudes to organ donation may affect the people commitment to do this, also, their knowledge and their faith plays an important role in their willingness to donate organs after death (Wakefield, Watts, Homewood, Meiser & Siminoff, 2010). Knowledge and attitudes of medical team about the organ donation is very important for planning to Increase the rate of organ donation (Salmani Nadoushan et al., 2014). Religious beliefs also play a role in determining attitudes to organ donation. Many religions have resisted the idea of organ donation (Rumsey, Hurford, & Cole, 2003).

In previous years many efforts have been made to raise public awareness about organ donation that as result, the number of Kidney donor increased to double. But because the number of recipients is increasing, most patients are placed on a waiting list more than 6 years (Donor Network of Croatia).

In England the process of organ donation is managed mainly by nurses and identification of potential donors is considered a nursing task. According to vital role that nurses play in the system of organ donation as promotion approach of potential donors for organ donation, explaining the process and getting consent, Nurses must receive sufficient training to understand the process in order to participate in satisfy the donors and their families (McGlade McClenahan, & Pierscionek, 2014).

Also the treatment team members are required to help patient and his family to make appropriate decisions in the final stages of life (Kent, 2007).Therefore, understanding their attitudes and knowledge can affect the tendency to organ donation which entails creation of effective education programs in universities (Tam, Suen, & Chan, 2012).

University training program must have basic information about the procedures and ethical issues related to organ donation to notify the medical staff in this domain (KY Chung et al., 2008). Despite the importance of the subject, few studies have assessed the knowledge, attitudes and practice of nurses regarding organ donation. So this study occurred to determine the level of professionalism and willingness of nurses as well as the effect of work experience on their attitudes about organ donation and its trends.

## 2. Materials and Methods

This is a descriptive-sectional study. In total 150 nurses employed in hospitals in 2013 were randomly selected. The methods and aims of the study were explained to them and ensure them that the individual information will be kept private and after they signed consent forms. Data collection tool was a multi section questionnaire, the first section included demographic information such as age, gender, education level and work experience. The second part consisted of standard questionnaire that was designed by KY Chung et al. (2008) which included 18 items that measured attitude and performance (answer: yes-no) and 15 items to measure the knowledge (answer: yes- no-I do not know) about organ donation.

### 2.1 Statistical Analysis

Data were analyzed using SPSS V15 software, by Chi-square test, Pearson test, t-test and ANOVA on a 95% confidence level.

## 3. Results

In this study, 18% of nurses were aged 18-24 years, 76% between 25-44 years and 6% of people between 45-64 years of age. 81.3% of samples were women (n = 122), 40.7% of nurses worked 1-5 years, 38% about 6-11 years, 13.3% about 12- 17 years and 8% more than 18 years. There was no significant difference between the two genders in receiving organ donation card (Table 1).

**Table 1:** Distribution of nurses having donation card according to gender differences

Organ donation card Gender		No	Yes	Total
**Male**	No	20	8	28
	Percent	71.4	28.6	100
**Female**	No	80	42	122
	Percent	65.6	34.4	100
**Total**	No	100	50	150
	Percent	66.7	33.3	100

***x2=0.351, p=0.553***				

However, difference between age groups in receipt of donation card was significant (Table 2).

**Table 2:** Distribution of nurses having donation card according to age groups

Organ donation card Age groups		No	Yes	Total
**18-24**	No	22	5	27
	Percent	81.5	18.5	100
**25-44**	No	70	44	114
	Percent	61.4	38.6	100
**45-64**	No	8	1	9
	Percent	88.9	11.1	100
**Total**	No	100	50	150
	Percent	66.7	33.3	100

***x2=6.08, p=0.048***				

Also there was a significant difference between the groups’ work experience in receiving organ donation card (Table 3).

**Table 3:** Distribution of nurses having donation card according to work experiences

Organ donation card work experience		No	Yes	Total
**1-5**	No	44	17	61
	Percent	72.1	27.9	100
**6-11**	No	34	23	57
	Percent	59.6	40.4	100
**12-17**	No	11	9	20
	Percent	55	45	100
**+18**	No	11	1	12
	Percent	91.7	8.3	100
**Total**	No	100	50	150
	Percent	66.7	33.3	100

***x2=6.68, p=0.083***				

The participants answer all questions about insights, performance (Table 4) and knowledge (Table 5).

**Table 4:** Distribution of nurses responding to attitude and performance questions

Questions		No	Yes
I support organ donation.	No	27	123
	Percent	18	82
I agree to donate my organs when I die.	No	27	123
	Percent	18	82
I have signed the organ donation card/filled in the organ donation form.	No	100	50
	Percent	66.7	33.3
I think the preservation of an intact body after death is important.	No	63	87
	Percent	42	58
I feel uncomfortable to think or talk about organ donation.	No	30	120
	Percent	20	80
I think the body will be disfigured when the organs are removed.	No	29	121
	Percent	19.3	80.7
I think there will be premature termination of medical treatment for registered organ donors.	No	33	117
	Percent	22	78
I think donating one’s organs adds meaning to one’s life.	No	28	122
	Percent	18.7	81.3
My family would object if I were to donate my organs.	No	55	95
	Percent	36.7	63.3
I know family members or close friends who have signed the organ donation card.	No	32	118
	Percent	21.3	78.7
I know people who have benefited or are in need of an organ transplant.	No	66	84
	Percent	44	56
I think live organ donation is better than cadaveric organ donation in solving the problem of organ shortage.	No	101	49
	Percent	67.3	32.7
I think it is convenient to register as an organ donor.	No	29	121
	Percent	19.3	80.7
I know where to obtain organ donation cards.	No	27	123
	Percent	18	82
I will agree to the donation of my family members’ organs.	No	59	91
	Percent	39.3	60.7
I am confident in approaching relatives of potential organ donors diagnosed brain dead and discussing issues related to organ donation with them.	No	124	26
	Percent	82.7	17.3
I am competent and have adequate knowledge in counseling patients on issues related to organ donation.	No	95	55
	Percent	63.3	36.7
I believe I have learnt enough about organ donation from the educational curriculum.	No	97	53
	Percent	64.7	35.3

**Table 5:** Distribution of nurses responding to knowledge questions

Questions		No	Yes
Malignancy is ALWAYS a contra-indication to cadaveric organ donation.	No	49	101
	Percent	32.7	67.3
The donor’s human leukocyte antigen MUST be identical to that of the recipient for any transplantation.	No	50	100
	Percent	33.3	66.7
The donor’s and recipient’s blood group MUST be identical.	No	51	99
	Percent	34	66
Organ transplant recipients are more prone to development of cancer after transplantation.	No	125	25
	Percent	83.3	16.7
Hepatitis B and C carriers can donate all of their solid organs except the liver.	No	45	105
	Percent	30	70
It is possible to transplant an adult liver into a paediatric patient.	No	115	35
	Percent	76.7	23.3
Increased risk of opportunistic infections is a complication common to all transplantations.	No	16	134
	Percent	10.7	89.3
In a brain-dead patient, all brain stem reflexes are absent.	No	63	87
	Percent	42	58
The heart can be beating in a brain-dead patient.	No	17	133
	Percent	11.3	88.7
A certified brain-dead registered organ donor will be immediately disconnected from mechanical ventilation support.	No	40	110
	Percent	26.7	73.3
More than 20% of the people on the renal transplant waiting list will receive an organ within a year.	No	107	43
	Percent	71.3	28.7
Registration of organ donors bears no age restriction.	No	96	54
	Percent	64	36
About 20 cadaveric livers are supplied each year.	No	97	53
	Percent	64.7	35.3
The organ donation rate in Iran is amongst the top 10 of the world.	No	103	47
	Percent	68.7	31.3
Having registered as an organ donor, consent from next-of-kin is still legally necessary for the removal of organs.	No	110	40
	Percent	73.3	26.7

The average attitude was 85.25±35.61 in women and 70.37±46.53 in men, which was significantly lower in men (p <0.05).The average score of practice in men and women was 34.43±47.71 and 29.63±46.53, respectively. The average score of clinical competence was 29.51±34.6 and 29.63±39.58, the average score of knowledge was 50.60±16.19 and 56.54±17.48 and the mean of factors influencing attitude and practices was 70.97±22.45 and 63.27±25.45. There was a significant difference respect to p-value of more than 0.05.

The mean attitude score of 18-24, 25-44 and 45-64 years old groups was 70.37±46.53, 85.09±35.78 and 77.78±44.09, respectively. However there was no significant difference between them (p>0.05).

The mean practice score of these groups was 18.52±39.58, 38.60±48.9 and 11.11±33.33, respectively. There was a significant difference between them (p<0.05) and show a better performance in the 25-44 years old group.

The mean clinical competence score of these groups was 33.33±39.22, 27.19±33.65 and 51.85±41.20, respectively. There was no significant difference between them (p>0.05).

The mean knowledge score of these groups was 55.56±19.66, 50.06±15.77 and 62.96±10.6, respectively. There was a significant difference between them (p<0.05).

The mean factors influencing attitude and practice score of these groups was 62.04±26.99, 71.49±22 and 63.89±23.94, respectively. There was no significant difference between them (p>0.05).

The results showed that the mean attitude score for those with 1-5 years of work experience was 80.33±40.08, for6-11 group was 84.21±36.79, for12-17 group was 80±41.04 and for +18 group was 83.33±38.92 that there was no significant difference (p>0.05)

The mean clinical competence score for these groups was 25.68±36.21, 26.32±31.96, 40±36.83, and 50±38.92, respectively, that there was no significant difference (p>0.05)

The mean knowledge score for these groups was 50.16±17.79, 49.82±14.59, 56.33±19.03 and 62.22±9.57, respectively, that there was no significant difference (p>0.05)

The mean factors influencing attitude and practice score for these groups was 67.49±23.11, 71.35±21.36, 68.75±29.23 and 70.14±23.69, respectively, that there was no significant difference (p>0.05)

According to the Pearson correlation test there was a significant direct relationship between attitudes and Factors influencing attitude and practice, but the relation between knowledge and attitude was significant and indirect (Table 6).

**Table 6:** Correlation between different aspects of the study

Pearson Correlations		Attitude	Action	Clinical competence	Knowledge	Factors influencing attitude and practice
**Attitude**	r	1	0.331	0.035	- 0.183	0.866
	p		0.000	0.673	0.025	0.000
**Action**	r	0.331	1	0.138	- 0.004	0.392
	p	0.000		0.092	0.963	0.000
**Clinical competence**	r	0.035	0.138	1	0.405	0.200
	p	0.673	0.092		0.000	0.014
**Knowledge**	r	- 0.183	- 0.004	0.405	1	- 0.145
	p	0.025	0.963	0.000		0.076
**Factors influencing attitude and practice**	r	0.866	0.392	0.200	- 0.145	**1**
	**p**	**0.000**	**0.000**	**0.014**	**0.076**	

In people who have had organ donation card, mean attitude, factors influencing attitude and practice score was significantly higher than those did not have donation card. However, there was no difference between clinical competence and knowledge score in tow groups.

Among those who think it is important to preserve the body after death, 33% had organ donation card, and 33% of those who did not have such thought, registered for donation card so this view had no effect on their performance.

36.3% of agree and 28% of disagree participant to donate their family member’s organs after death, had organ donation card. There was no significant difference between two groups.

47.2% of those declared trained about organ donation through university curriculum and 25.8% of those did not receive adequate training, had organ donation card. This difference was significant and training can be effective on organ donation.

None of those who thought that “a person had an organ donation card, will not receive full medical treatment” had donation card. So the training can be effective on organ donation preference.

96.6% of those have any donation card believed it is not easy to register for organ donation and 100% of them did not know where receive such card.

Family members of 24% of those had organ donation card and 43% of others, agreed to donate their organs. There was a significant difference between two groups, it means that family members insight can effect on vision about organ donation.

## 4. Discussion

The main objective of this study was to assess the knowledge, attitude and practice of nurses toward organ donation. Results showed that attitudes are related to the amount of knowledge but it expected to be negative and significant. Also people with higher attitude score have better practice in the matter of organ donation.

Tam et al. (2012) did a study to determine the knowledge, attitude and commitment of nursing students on 362 students. A total of 40.6% of them were registered for organ donation. The mean score of the participant’s knowledge was 23.7±2.9 and the mean score of their attitudes was 70.2±7.7. About 3.7% of students stated that received several propagandistic advertisement about organ donation through public media such as television, radio, newspapers, magazines, Internet web pages and other media, while in the present study, 33.3% of participants had organ donation card and 35.3% trained about organ donation by the university and hospital’s curriculum. Stadlbauer et al. (2013) results showed that their primary source of information was media, friends, family, newspapers and seminars. About 44.9% stated their willingness to donate their organs and 77.8% were agreed to encourage people toward organ donation.

In Georgiadou et al. (2012) research that evaluate the different factors related to the Egyptian’s willingness to donate organs on 2263 people, about 3.8% of participants were registered for organ donation. Almost half of them (48.3%) would receive a membership card, and 49.1% expressed willingness to donate organs of their relatives, but 55.7%of theme expressed concern about organ removal and transfer procedures. They believed that organ donation is a sin. Women tend to receive organ donation card much more than men. Most participants stated their information resources were mass media (70.6%) and magazines (46.2%), also treatment groups (33.6%) played a main role in their awareness in this area (Georgiadou et al., 2012). In our study 60.7 % of people expressed their willingness to donate organs of their relatives. Also, no significant differences were observed between the genders to receive donation card.

Zambudio et al. (2009) conducted a study on 305 nurses to analyze their attitudes toward organ donation and the factors determining these attitudes. About 63% want to donate their organs. Factors influencing their attitudes about organ donation included a favorable attitude toward family’s organ donation (p <0.001), knowing the concept of brain death (p <0.005), the definition of organ donation in the family (p = 0.001), favorable attitudes toward autopsy (p = 0.006), the fear of mutilation of the body (p <0.001), parental attitudes toward organ donation (p <0.001), and ultimately religious affiliation (p = 0.009). Also in this study, there was a significant and negative relationship between knowledge and attitudes (P <0.05 and r = -0.183) and a significant positive correlation between attitude and practice (Factors influencing attitude and practice) (P <0.05). In Salmani Nadoushan et al. (2014) study, about 78% agreed to donate organs after death and 68% had donation card. Also 78% had a positive attitude to receive organ donation card.

In another research 44.8% of participants reported that they have sufficient knowledge about organ donation and 40.1% have enough knowledge about organ transplantation. They stated the most important sources of information include mass media (72.1%).Willingness to donate their organs and their relatives was seen 58.4% and 39.9%respectively. Donation of living organ was more acceptable than cadaver’s organs (74.6%). Among the participants only 1.2% received organ donation card (Bilgel, Sadikoglu, & Bilgel, 2006). In another study, 46.8% of participants had organ donation card and 58% were willing to receive card and 13.8% were reluctant to do so (McGlade et al., 2014). In the present study, 82% and 60.7% were agreeing to donate their own and relatives’ organs, respectively.About36.7% stated that they would also counsel patients and their knowledge is sufficient in this domain.

A similar study on 262 medical students was conducted to assess the attitudes of physician students about organ donation. The results showed that medical students have a positive attitude about organ donation (4.34±0.46). Participants were also more inclined to donate their own organs (85%), not relatives (49.2%). There was no relationship between age, gender, education level and attitudes to organ donation (Sanavi, Afshar, Lotfizadeh & Davati, 2009). Another study, in most cases, participants orally and in writing stated that honor relatives regarding organ donation (Jernigan et al., 2013).

## 5. Conclusions

In this study, the mean attitude in men was significantly lower than women (P <0.05), but there was no relationship between attitude and age.

Researches show that the individual’s level of education, religious and attitude would be related to their commitment in organ donation. Because of effect of these factors on people’s willingness to donate organs, it seems that training and educational tools should be applied to improve employees’ attitudes towards this issue. Also findings highlight the need for training programs for treatment team and empowering them in all matters relating to donations of organs. Since the medical team have the most important guideline to promote activities related to organ donation, it seems educational curriculum should be involved this issue to change students behavior.
